# A Plausible Mechanism for the Iridium-Catalyzed Hydrogenation of a Bulky *N*-Aryl Imine in the (*S*)-Metolachlor Process

**DOI:** 10.3390/molecules27165106

**Published:** 2022-08-11

**Authors:** Amanda L. Kwan, Robert H. Morris

**Affiliations:** Department of Chemistry, University of Toronto, Toronto, ON M5S 3H6, Canada

**Keywords:** asymmetric catalysis, density functional calculations, hydrogenation, iridium, reaction mechanisms

## Abstract

The hydrogenation of *N*-(2-ethyl-6-methylphenyl)-1-methoxypropan-2-imine is the largest-scale asymmetric catalytic process for the industrial production of agrochemical (*S*)-metolachlor. The challenging hydrogenation across the sterically crowded carbon–nitrogen double bond was achieved using a mixture of [IrCl(COD)]_2_, (*R*,*S_Fc_*)-Xyliphos, NBu_4_I and acetic acid. Acetic acid was critical in achieving excellent productivity and activity. Despite its industrial significance, a mechanism that explains how the sterically hindered bond in the imine is reduced has yet to be proposed. We propose a plausible proton-first, outer-sphere mechanism based on density functional theory calculations that is consistent with the experimentally observed activity and the enantioselectivity of the industrial process. Key findings include transition states involving acetate-assisted dihydrogen splitting, and a hydride transfer from a five-coordinate iridium trihydride directed by a C-H∙∙∙Ir interaction. This article was submitted to a Special Issue in honor of Professor Henri Kagan.

## 1. Introduction

Chiral amines are a prevalent motif in the pharmaceutical and agrochemical industries, thereby motivating interest in the development of synthetic routes leading to enantiopure amine products [1,2,3,4]. One of the few ways that this chiral center can be installed is through the metal-catalyzed asymmetric reduction in unsaturated imine precursors using hydrogen gas (H_2_). Catalysts that facilitate asymmetric direct hydrogenation (ADH) across the carbon–nitrogen double bond (C=N) are highly coveted due to their high atom economy. However, these catalysts are often limited to a small set of substrates, and imine hydrogenation, in general, remains underexplored and less understood in comparison to olefin and ketone hydrogenation.

**Scheme 1:**
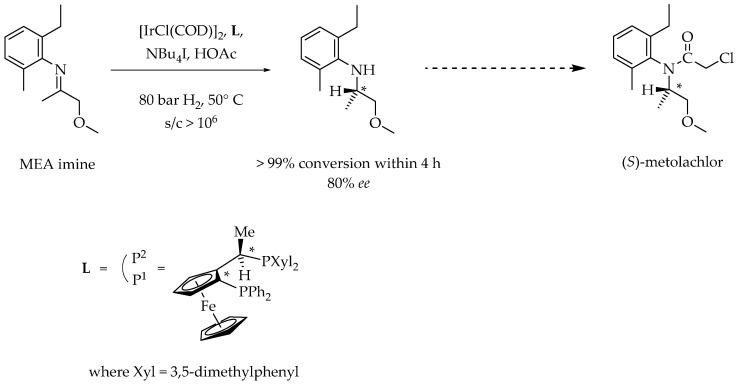
Industrial synthesis of agrochemical (*S*)-metolachlor via asymmetric hydrogenation of the MEA imine catalyzed by an iridium-(*R,S_Fc_*)-Xyliphos catalyst.

Many catalysts used in asymmetric imine hydrogenation are based on iridium. The most notable example is the Ir-Xyliphos system developed by Ciba-Geigy in the 1990s for the production of (*S*)-metolachlor, the active ingredient in Syngenta’s Dual Magnum herbicide. The reaction, shown in Scheme 1, is the largest-scale asymmetric hydrogenation process, yielding more than 10,000 tons per year of enantioenriched amine from *N*-(2-ethyl-6-methylphenyl)-1-methoxypropan-2-imine (MEA imine) [5,6,7,8,9,10,11,12]. The catalyst is generated in situ using a mixture of [IrCl(COD)]_2_ (COD = 1,5-cyclooctadiene), Xyliphos ligand (**L**), iodide salts and acid as additives. Tetrabutylammonium iodide (NBu_4_I) and acetic acid (HOAc) are commonly reported as the source of iodide and acid, respectively. **L** belongs to the Josiphos family of chiral diphosphine ligands with a ferrocenyl moiety in the backbone. The reaction, performed using 80 bar H_2_ at 50 °C, exhibits excellent activity with initial turnover frequencies (TOFs) exceeding 1.8 × 10^6^ h^−1^, excellent productivity with turnover numbers (TON) exceeding 2 × 10^6^ and good selectivity with an enantiomeric excess (*ee*) of 80%. The iodide and acid additives were critical to the success of the reaction. In the breakthrough discovery, acetic acid was used as the solvent and the activity was found to enhance ten-fold [10]. In the optimized technical process, acetic acid was used as an additive instead of a solvent.

**Figure 1:**
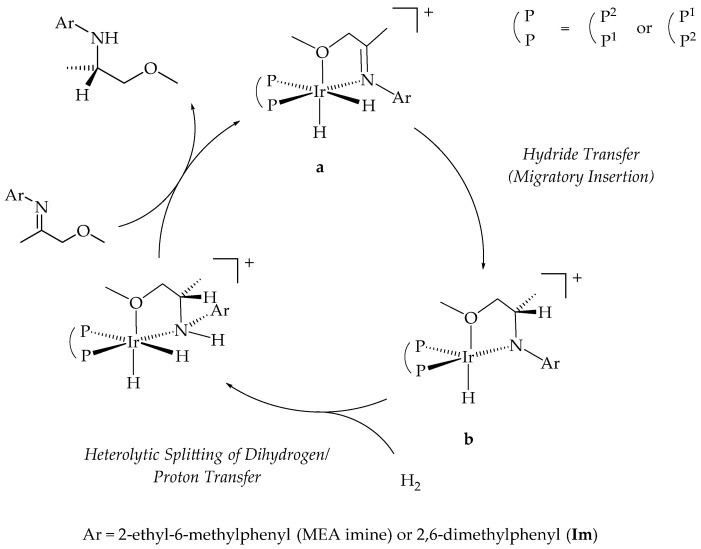
Inner-sphere mechanism with substrate coordination to iridium (previous works [11,12]).

Despite the industrial and scientific significance of the (*S*)-metolachlor process, the system is not well-defined. The “magic mixture” consisting of iridium, **L**, iodide and acid generates several iridium complexes, of which at least one is a precursor to the catalytically active species [12]. Previous investigations postulated the occurrence of an inner-sphere mechanism, in which the substrate is coordinated to Ir in one of the intermediates, in a κ^2^-fashion through the N and O functionalities of the MEA imine [11,12] (Figure 1); other inner-sphere systems have also been reported [4,13,14]. Togni and coworkers identified, with NMR, the formation of four diastereomers of [IrH_2_(κ^2^-**Im**)((*S*,*R_Fe_*)-Xyliphos)]^+^ under a low pressure of dihydrogen, but in the absence of the other additives required for catalysis [12]. In their studies, the imine used had the simpler 2,6-dimethylphenyl substituent on the nitrogen (imine denoted **Im** in the current work), and the Xyliphos ligand used had the opposite enantiomer of the one used in the industrial process. They speculated that the nucleophilic attack on the coordinated imine by the hydride *trans* to phosphorus in one diastereomer would lead to the amine product. We were unable to find a viable inner-sphere mechanism of this type using density functional theory (DFT) calculations (see below). To our knowledge, DFT calculations have not yet been utilized to investigate the (*S*)-metolachlor system.

**Figure 2:**
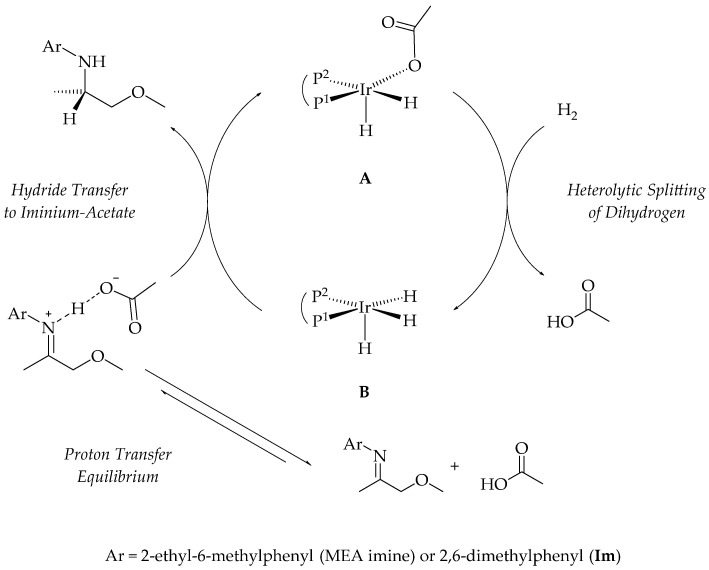
Outer-sphere mechanism without κ^2^-substrate coordination to iridium (this work).

In the years since the inner-sphere mechanism was proposed, outer-sphere mechanisms have been proposed for the hydrogenation of *N*-phenyl imines [3,15,16,17,18,19], *N*-heterocyclic imines [19,20,21] and even less bulky *N*-methyl imines [18]. Due to the sterically crowded C=N bond, we opted to investigate an outer-sphere mechanism for the ADH of the MEA imine (Figure 2). In this alternative mechanism based on DFT, the substrate is first protonated (converted to iminium) before the outer-sphere hydride transfer from a neutral Ir(III) complex to generate the (*S*)-amine product. The five-coordinate Ir-trihydride intermediate is regenerated through heterolytic H_2_ splitting. In this mechanism, the catalyst confers selectivity to the substrate upon hydride transfer without the need for the substrate to coordinate to the metal, other than an intermolecular C-H∙∙∙Ir interaction. This interaction may play a role in stabilizing the transition state and directing the enantioselectivity of the reaction [22]. We propose that the xylyl groups on the Xyliphos ligand prevent the catalyst from dimerizing to a stable dinuclear species [Ir_2_(μ-H)_3_L_n_]^+^, a common motif in iridium hydride chemistry that is often proposed as a deactivation pathway for hydrogenation catalysts [23,24,25,26,27,28]. Indeed, bulky substituents for coordinating phosphines have been used as a strategy against catalyst deactivation [29].

A mechanism for the (*S*)-metolachlor imine hydrogenation process should be consistent with experimental observations and account for the roles of the iodide salt and acetic acid additives. Catalyst precursors based on neutral Ir(I) complexes (such as [IrCl(COD)]_2_) are often enhanced with or require a halide additive for activity, usually iodide, but, sometimes, chloride or bromide [3,30,31]. We assumed, with some computational evidence, that iodide is a large, weakly coordinating anion that permits the formation of five-coordinate intermediates. In the (*S*)-metolachlor system, the catalyst is operational without an acid additive (that is, iodide only); however, acetic acid enhances the activity to meet industrial targets and even improves the selectivity by 5–6% [10]. The use of a Brønsted acid additive has been reported several times with Josiphos systems [31,32,33,34] (and other hydrogenation systems reviewed elsewhere [2,35,36,37]). There exists a mechanistic precedence for Brønsted conjugate bases participating in C-H [38,39] and H-H [40,41,42] bond activation, where a change in the coordination mode is possible, such as with formate or acetate. In general, the roles of additives in catalysis are not well understood, since the types and effects of additives can vary across similar systems. To summarize, we took into account the following four considerations:

**I. Activity**: The energetic span of the catalytic cycle should be overcome at 50 °C.

**II. Selectivity**: The mechanism should predict the correct enantiomer (*S*) with an *ee* of approximately 80%. Therefore, the energetic span of the (*R*)-pathway should be greater than the energetic span of the (*S*)-pathway by approximately 1.3 kcal mol^−1^.

**III. The role of acetic acid**: Catalytic activity, albeit low, was reported even for systems without acetic acid or acetate (HOAc/^−^OAc). However, activity was enhanced with the addition of acetic acid, and some accounts of the (*S*)-metolachlor process reported the use of sulfuric acid as the acid additive [2,3,10,11,12]. Therefore, a reasonable mechanism should be assisted by, but not dependent on, the presence of acetic acid.

**IV. The role of iodide**: Iodide is a weakly coordinating anion that allows for the formation of coordinatively unsaturated catalytic intermediates.

Herein, we report a plausible mechanism supported by DFT that is consistent with the above considerations. By investigating one of the most efficient industrial catalysts, we are optimistic that a mechanistic understanding of the (*S*)-metolachlor process could expand our toolbox for catalyst design.

## 2. Results

### 2.1. Overview

**Figure 3:**
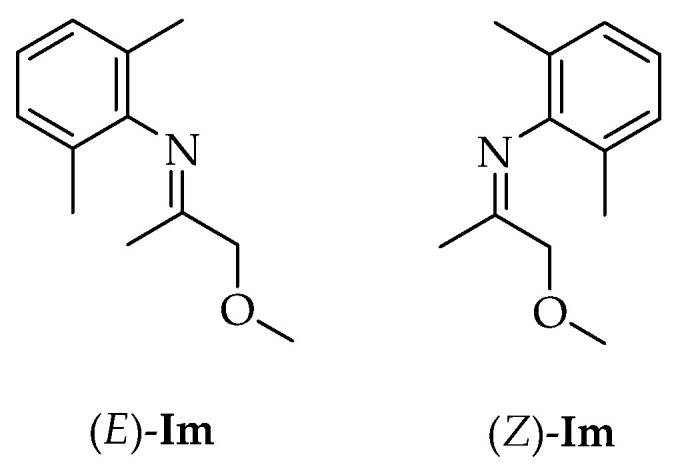
Stereoisomers of **Im**. *E/Z* is 86:14 in acetic acid (calculated) and neat (experimental).

The simpler but similar imine *N*-(2,6-dimethylphenyl)-1-methoxypropan-2-imine **Im** (Figure 3) was used as the substrate for the study. We reasoned that the ethyl group on the *N*-aryl group was not essential for calculations and could be substituted with the methyl group instead. (One of the methyl groups was considered relevant to the mechanism, as discussed in Section 2.4). The hydrogenation of **Im** was reported to yield an 87% *ee* using a Josiphos ligand, although no further details regarding the conditions, productivity, activity or catalytic system were specified [10].

In acetic acid, **Im** exists as an equilibrium of *E* and *Z* isomers favoring (*E*)-**Im**, which puts the sterically bulky *N*-aryl and ether groups on opposite sides of the C=N bond. The *E*/*Z* ratio was calculated to be 86:14 in acetic acid (refer to “Isomerization Calculation” including Scheme S1 and Table S1 in Supplementary Materials), which was consistent with experimental observations of the isolated imine [11,12]. It could reasonably be assumed that isomerization was fast, and that the *E*/*Z* ratio remained constant [15,43,44].

**Figure 4:**
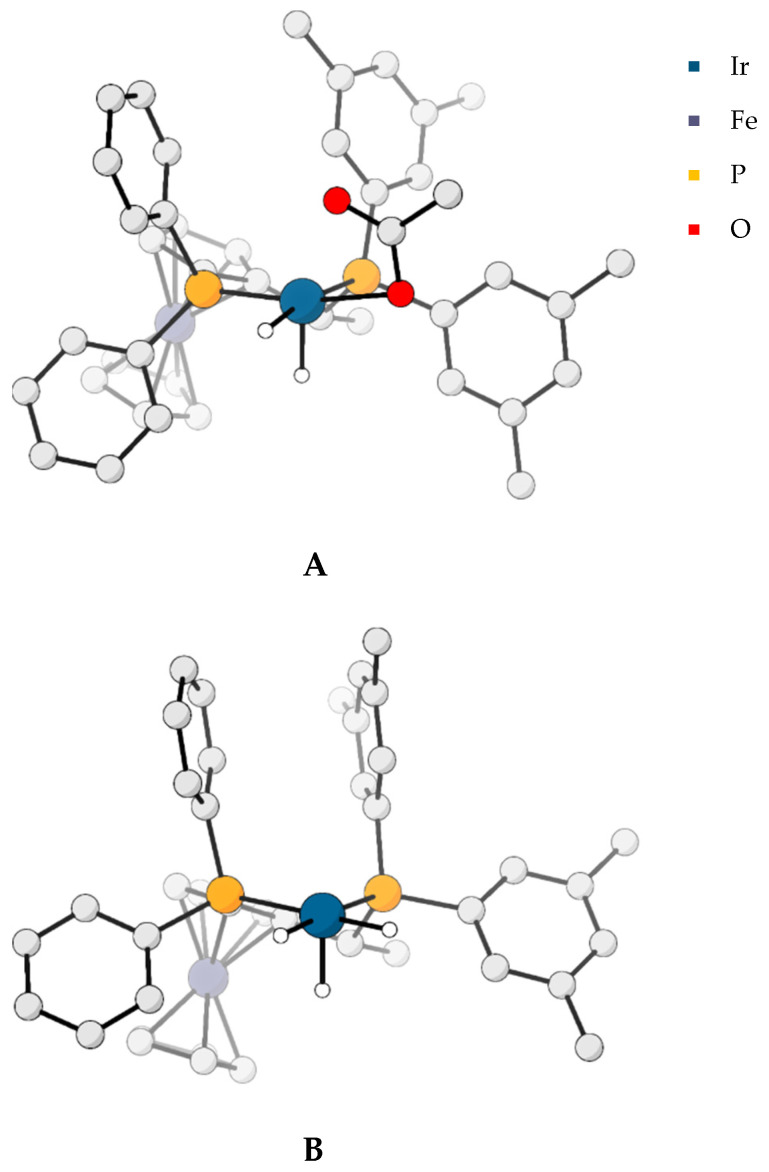
Iridium dihydride (**A**) involved in dihydrogen splitting and iridium trihydride (**B**) involved in hydride transfer. C-H hydrogens were omitted for clarity.

**Figure 5:**
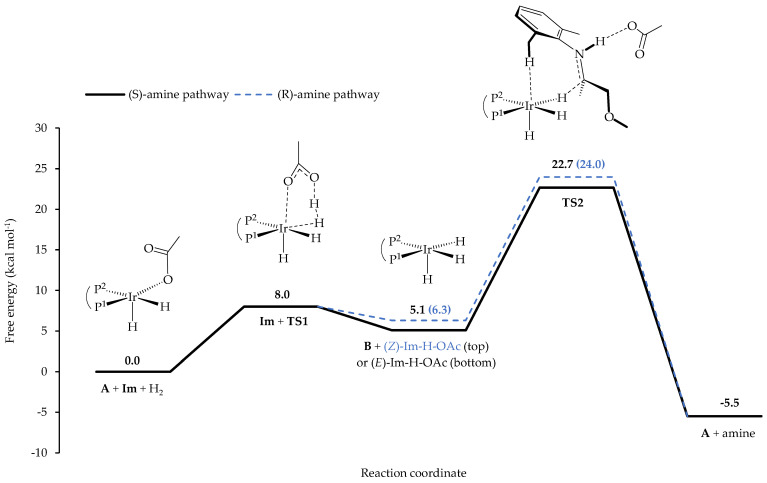
Reaction coordinate diagram and transition states for proposed hydrogenation mechanism in acetic acid.

As a consequence of consideration II we first elucidated the enantioselective step: the hydride transfer to an iminium carbon. An iridium trihydride **B** (Figure 4) delivered promising results. Others have proposed that iridium trihydrides are active in other polar bond hydrogenation processes [3,20,45,46,47,48,49,50,51,52]. We also investigated a complex of the form IrH_2_LOAc as a potential candidate for hydride transfer, but the barrier was found to be too high and, thus, was not discussed in the main text (**TS^V^** in Supplementary Materials Figure S6). Working backward, related complexes of [IrH_2_L]^+^ were explored to determine how **B** was regenerated after the hydride transfer. We proposed the proton-first, outer-sphere hydrogenation of **Im** via the catalytic cycle shown in Figure 2. The reaction coordinate profile for this mechanism is shown in Figure 5.

Lastly, in Section 2.7, we briefly discussed calculations pertaining to the intermediates of the inner-sphere mechanism shown in Figure 1.

### 2.2. Heterolytic Splitting of Dihydrogen

**Figure 6:**
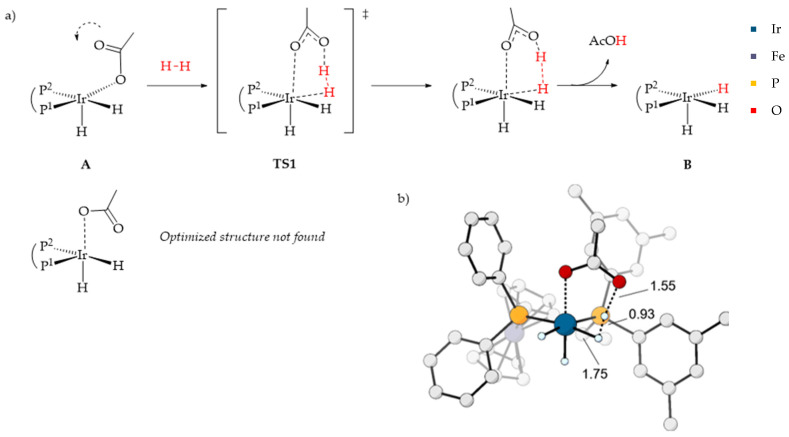
(**a**) “Catcher’s mitt” mechanism by iridium-bound acetate, which “catches” the proton from heterolytic splitting of H_2_. (**b**) Structure of **TS1** with relevant atomic distances reported in Å. C-H hydrogens were omitted for clarity.

The reaction coordinate diagram in Figure 5 begins with iridium dihydride **A**. Ground-state optimizations showed that **A** was preferentially formed over the isomer shown in Figure 6a with acetate *trans* to a hydride (attempts to calculate a low-energy structure of this isomer resulted in the acetate reverting to the binding site shown in **A**). However, the energetic barrier of isomerization (ΔG^‡^_1_ = 8.0 kcal mol^−1^) could be overcome in the presence of H_2_. Like a “catcher’s mitt,” acetate swung open to the other binding site, and, in the process, heterolytically split H_2_: the proton to the acetate, and the hydride to the iridium to form the iridium trihydride **B** via transition state **TS1** in Figure 6b. An animation (GIF) of **TS1** is included in the Supplementary Materials. An intramolecular dihydrogen bond interaction (Ir-H∙∙∙H-O) could be observed in **TS1,** as demonstrated by the atomic distances shown in Figure 6b, which was previously reported in other complexes [53,54,55].

Once the acetate was protonated, it was easily displaced from the coordination sphere as acetic acid, an even a weaker ligand, which could be substituted with other species in solution. Thus, ΔG^‡^_1_ encapsulated the energy required for acetate to switch binding modes and cleave the H-H bond. This energy barrier was much less than that of the hydride transfer step discussed later, and so was a reasonable pathway. We also explored an alternative mechanism, in which acetate was not coordinated to iridium and split H_2_ in the outer sphere (**TS^III^** in Supplementary Materials Figure S5).

### 2.3. Proton Transfer Equilibrium

Herein, we report a proton-first mechanism as opposed to a hydride-first mechanism. In solution, imine **Im** and acetic acid existed as an adduct Im-H-OAc (shown in Figure 2). The hydrogen bond interaction between the acetate and **Im** served to increase the electrophilicity of the imine/iminium carbon for the hydride attack. Further discussion about the Im-H-OAc adduct is presented in the Supplementary Materials (“Proton Transfer Equilibrium” including Figure S3).

### 2.4. The Enantioselective Step: Hydride Transfer to Iminium-Acetate

As previously mentioned, our search for a plausible mechanism began with elucidating the hydride transfer mechanism and catalytic species in order to satisfy consideration II. This proved to be challenging with many iridium–hydride complexes that were generated from the catalyst mixture. Indeed, many species were experimentally observed in the catalyst mixture, where one or some complexes were highly catalytically active [12]. For complexes with the formula [IrH*_x_*L]*^z^* (*x* = 2, 3; *z* = 0, 1), there were “four choose *x*” configurations assuming a pseudo-octahedral geometry (four is the number of possible positions after the bidentate chelation by L)—i.e., four IrH_3_L complexes and six [IrH_2_L]^+^ complexes. Moreover, for every iridium–hydride complex, there were *x* possible hydride transfer pathways. This did not yet include acetate-, chloride- or iodide-coordinated complexes, for which there were as many as 4! = 24 configurations per formula if all positions were occupied by different ligands.

**Figure 7:**
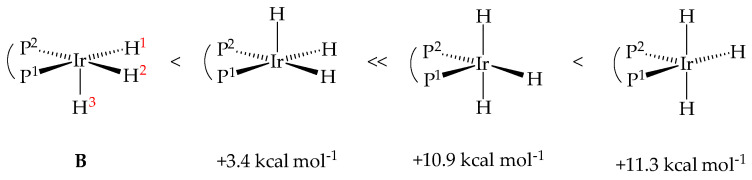
IrH_3_L configurations and their free energies relative to **B** in acetic acid.

As with most systems, it was impractical to investigate all *possible* hydride transfer pathways from every iridium complex. Instead, we turned our attention to some *probable* pathways based on previously reported systems and intuitions. Since neutral hydrides are more nucleophilic than cationic iridium hydrides, and complexes of IrH_3_L have established precedence in polar bond hydrogenation [3,20,45,46,47,48,49,50,51,52], we investigated configurations of IrH_3_L as candidates for hydride donation (Figure 7). As expected, trihydride structures with *trans* hydrides had relatively high free energies. The lowest energy configuration was **B,** with P^1^ representing the PPh_2_ donor and P^2^ the Pxylyl_2_ donor (Scheme 1), which we proceeded to investigate.

**Figure 8:**
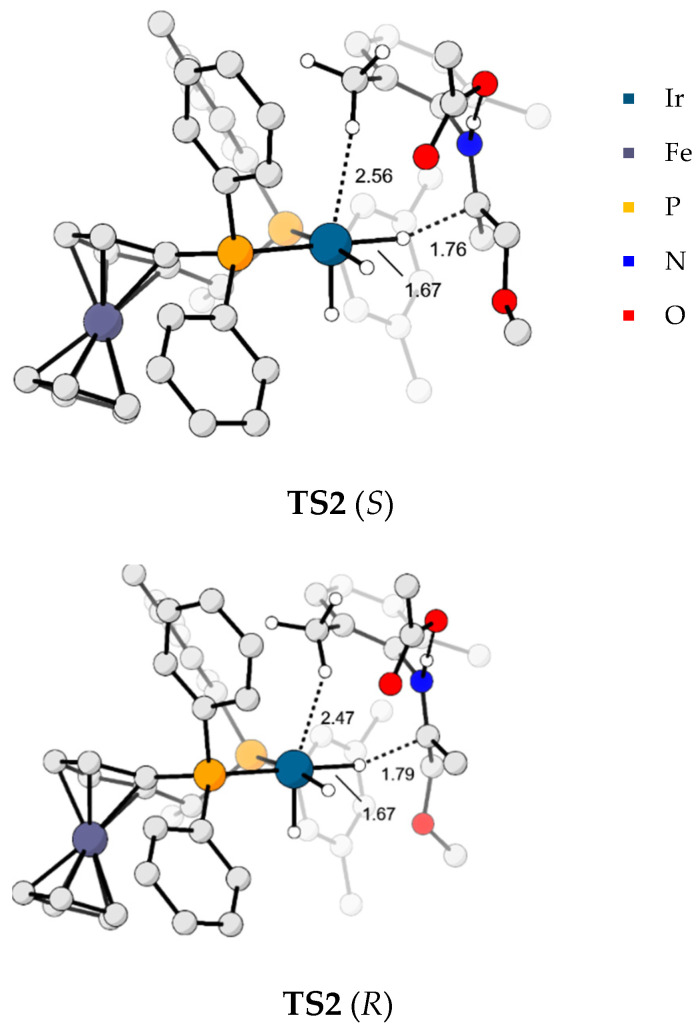
Prochiral structures of **TS2** in acetic acid with relevant atomic distances reported in Å. Most C-H hydrogens were omitted for clarity.

Complex **B** had three potential hydride transfer pathways (delivery of H^1^, H^2^ or H^3^ labeled in Figure 7). The substrate could not be positioned so that H^3^ could approach the iminium carbon due to steric clashes between the *N*-aryl group on the iminium and the ferrocenyl group on the ligand backbone. However, hydride transfers of H^1^ and H^2^ offered promising leads because the *N*-aryl group could be positioned over the vacant site of the complex. In this way, we found pro-(*S*) and pro-(*R*) transition states **TS2** from the hydride transfer of H^1^ (Figure 8). An animation (GIF) of **TS2** (*S*) is included in the Supplementary Materials. The hydride transfer of H^2^ was ruled out, following some preliminary calculations (**TS^VI^** and **TS^IX^** in Supplementary Materials Figure S6).

In Table 1, we reported activation energies that predicted an *ee* of 81% (*S*) in acetic acid. Our mechanism predicted the correct enantiomer and selectivity. Interestingly, the results were sensitive to the implicit solvent used—in tetrahydrofuran, which had a dielectric constant of 7.6 compared to 6.2 for acetic acid, the iminium carbon-hydride distances decreased by 0.1 Å, the energetic barriers for both pathways increased and the predicted *ee* increased to 98% (*S*). The dielectric constant of acetic acid was low due to the formation of a stable hydrogen-bonded dimer. It should be noted here that no explicit solvents were used apart from an acetate molecule in the iminium-acetate adduct, and that the solvation model based on density (SMD) represented implicit solvents with a set of properties (dielectric constants, hydrogen bond acidity/basicity constants, indices of refraction, etc.) [56]. Thus, the differences arising from acetic acid versus tetrahydrofuran transition states arose from differences in these properties. A previous study also observed that with outer-sphere imine hydrogenation mechanisms, the enantioselectivity was considerably more sensitive to solvent and temperature effects due to the flexibility of the substrate, since it was not coordinated to iridium [16]. Evidently, the former effect was observed here.

Nonetheless, all of the structures exploited an intermolecular C-H∙∙∙Ir interaction between *N*-xylyl methyl hydrogen and iridium; reported atomic distances ranged between d(Ir-H_xyl_) 2.4 and 2.6 Å, consistent with an overlap of their van der Waals (vdW) spheres [57]. The distance was longer than the Rh-H-C distances of 1.9 and 2.2 Å calculated for the ethane sigma complex [[Rh(PONOP)(η^2^-H_3_CCH_3_)][BAr^F^_4_] [58,59]. This interaction may play a role in stabilizing the transition state and directing enantioselectivity. This was consistent with the well-known propensity of iridium to activate C-H bonds [19,57,60,61,62] and was reminiscent, in particular, of a *meta*-selective C-H borylation system [63]. In the MEA imine, which had an asymmetric *N*-aryl group, we proposed that this function was assumed by a methyl hydrogen and iridium, while the ethyl group was held away from the iridium.

**Figure 9:**
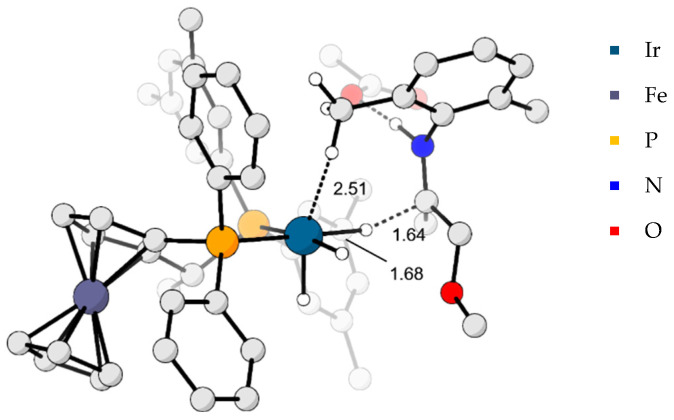
Alternative pro-(*S*) transition state **TS2** (*S*’) with hydride transfer to the higher energy (*Z*)-iminium-acetate adduct with relevant atomic distances reported in Å. Most C-H hydrogens were omitted for clarity.

Enantioselectivity did not appear to be determined by intramolecular steric effects, i.e., the imine or iminium-acetate conformation (*E* or *Z*). In **TS2** (*S*), hydride H^1^ attacked the electrophilic carbon of the more stable (*E*)-iminium-acetate adduct to form the (*S*)-product. In **TS2** (*S’*) in Figure 9, hydride H^1^ attacked the electrophilic carbon of the less stable (*Z*)-iminium-acetate adduct to form the same product. **TS2** (*S’*) had the same free energy barrier as **TS2** (*S*) relative to the starting state, despite the higher energy intermediate. Since imine mixtures with small *E*/*Z* ratios can still yield a high *ee* [3,15,43], the intermolecular steric effect, i.e., between the iminium carbon substituents (-CH_2_OCH_3_ versus -CH_3_) and the ligand P^2^ xylyl groups, seemed to be the enantioselective determining factor. In **TS2** (*R*), the bulkier substituent -CH_2_OCH_3_ was held near one of the bulky xylyl groups on the ligand. The proximity of a substrate methoxy hydrogen to a xylyl methyl hydrogen forced the xylyl methyl group to rotate away from the methoxy group. Without this distortion, the methoxy hydrogen entered within the vdW radius of the xylyl methyl hydrogen. The xylyl methyl group rotation on the ligand may play a role in increasing the activation barrier of the pro-(*R*) transition state with respect to the pro-(*S*) transition states **TS2** (*S*) and **TS2** (*S*’), where the less bulky -CH_3_ group on the substrate did not induce the xylyl methyl rotation. The distortion, although subtle, caused the pro-(*R*) transition state to be higher in energy.

### 2.5. Deactivation

As previously discussed, the formation of [Ir_2_(μ-H)_3_L_n_]^+^ is a known deactivation pathway for hydrogenation catalysis. Modeling of these structures using DFT was too costly. We speculated that Pxylyl–Pxylyl intermolecular interactions would interfere with dimer formation.

### 2.6. Revisiting the Considerations

Our mechanism correctly predicted the (*S*)-amine product, although the predicted *ee* depended on the medium used in the implicit solvation model. Accounts of the technical process reported that the reaction was performed neat (no solvent in the bulk imine) [9,11]. Many of the constants used to define solvents in the SMD model (such as the dielectric constant) were not known or reported for the industrially relevant imine, so simulating exact conditions used in the technical process was not possible. Our results were primarily reported using acetic acid as the implicit solvent to simulate the breakthrough discovery [10]. Nonetheless, our mechanism proposed pathways with energetic spans of less than 25 kcal mol^−1^ for the forward reaction, which is feasible even in nonoptimal conditions (at lower temperatures, pressures and in acetic acid instead of neat). Therefore, we were satisfied with the fulfillment of considerations I and II.

**Figure 10:**
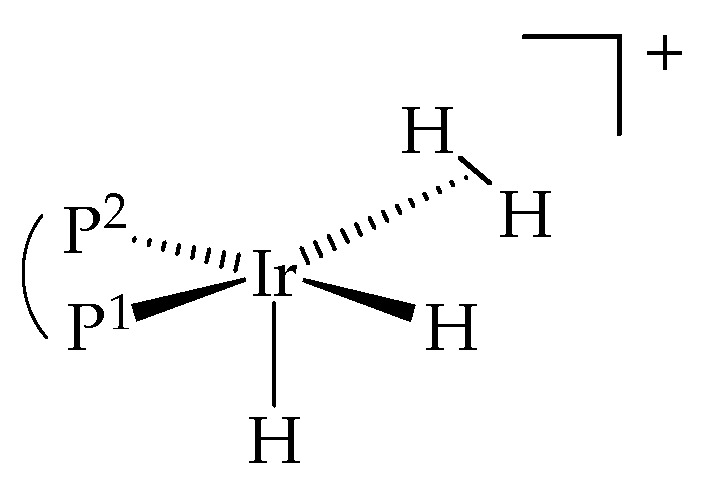
Iridium dihydrogen–dihydride complex, a proposed intermediate in an acetate-free mechanism.

In the absence of acetic acid, an outer-sphere hydrogenation mechanism was still possible if the role of acetate described here was assumed by another base (iodide, chloride or another equivalent of imine), satisfying considerationIII. We hypothesized that these mechanisms were plausible, but proceeded with higher energetic barriers, which would explain why the acetic acid drastically improved the outcome of the technical process by assisting in the “catcher-mitt” dihydrogen activation [10]. It is possible that H_2_ coordinated first to the iridium, giving a dihydride–dihydrogen complex that was deprotonated by the imine to yield the iridium trihydride species (the optimized structure of the dihydride–dihydrogen complex was calculated and is shown in Figure 10) in lieu of the acetate-assisted heterolytic splitting of H_2_ and proton transfer described here. In the absence of acetate, the proton would transfer to the imine followed by the hydride transfer to iminium (**TS^VII^** and **TS^VIII^** in the Supplementary Materials Figure S6), similar to other previously reported outer-sphere mechanisms from iridium-trihydrides [20].

In the results reported here, iodide was treated as a weakly or noncoordinating counterion based on a sample calculation, in which the iridium–iodide distance exceeded standard iridium–iodide bond lengths (refer to “Iridium-Iodide Distances” including Figures S1 and S2 in the Supplementary Materials). We proposed that the role of iodide was to permit the formation of the coordinatively unsaturated iridium trihydride (five-coordinate octahedral geometry), fulfilling consideration IV.

### 2.7. Off-Cycle Imine Coordination

**Figure 11:**
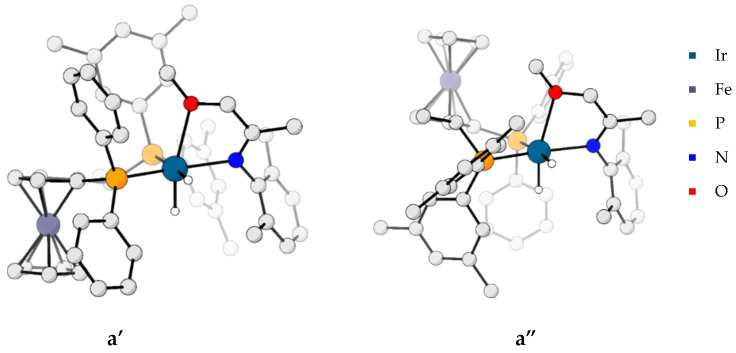
Pro-(*S*) diastereomers of complexes **a,** proposed intermediates in the inner-sphere mechanism that were prepared in previous work [11,12]. C-H hydrogens were omitted for clarity.

In addition to the outer-sphere mechanism we proposed, we investigated some structures of [IrH_2_(κ^2^-**Im**)((*S*,*R_Fe_*)-Xyliphos)]^+^ described previously [12] as the mirror images that might be involved in the inner-sphere mechanism shown in Figure 1 with imine (**a**) and amide (**b**) intermediates. The structures of two pro-(S) diastereomers of complexes **a** (denoted as **a’** and **a”**) with the imine coordinated to iridium were optimized (Figure 11). Attempts to optimize their respective structures with the amide coordinated to iridium (complexes **b** of Figure 1) and locate transition states modeling hydride transfer/migratory insertion were unsuccessful.

In previous investigations, the diastereomeric mixture of complexes **a** was formed in the absence of acid and halide salts, and characterized with NMR spectroscopy [11,12]. The free energy of cationic complex **a’** was lower in energy than the combined free energies of its precursor cationic iridium dihydrides and **Im** by 18.8 kcal mol^−1^. While the formation of complex **a’** was thermodynamically favorable, and there was excess **Im** in the system, the kinetic barriers of the **Im** association to cationic iridium dihydrides were presumably higher than the kinetic barriers of acetate association due to the larger size and neutral charge of the imine or cationic charge of the protonated imine. We proposed that complexes **a** were off-cycle equilibrium species in the absence of acid and halide salts that decreased the activity of the catalyst. The formation and stability of the dihydride complexes [IrH_2_(κ^2^-**Im**)((*S*,*R_Fe_*)-Xyliphos)]^+^ **a** under 2-bar H_2_ and their characterization with NMR spectroscopy might indicate that they were not within the catalytic cycle toward amine formation.

## 3. Discussion

A proton-first, outer-sphere mechanism, in which the sterically crowded substrate only bound to iridium via a C-H interaction, was shown to be a plausible mechanism for the asymmetric hydrogenation of **Im**. Many catalyst configurations and reaction pathways were eliminated from further study in preliminary calculations (more information is provided in Supplementary Materials “Other Structures and Pathways” including Figures S4–S6 with thermochemical values reported in Table S4) and the inner-sphere mechanism intermediates were briefly investigated; however, all possible mechanisms were not exhausted. An investigation into these mechanisms, the use of a larger basis set and the use of explicit solvent molecules would certainly improve our comprehensive understanding of this catalytic system, but are too costly at this time.

In addition to our report of a novel mechanism for the largest-scale asymmetric hydrogenation process, we are optimistic that mechanistic insights from our study could be used towards the development of new catalysts. In particular, our group hopes to extend the results of this study to synthesize earth-abundant metal catalysts that can perform asymmetric hydrogenation for industrially relevant imines, including the replacement of the iridium–Xyliphos catalyst with a more sustainable and inexpensive alternative for (*S*)-metolachlor production.

## 4. Materials and Methods

All calculations herein were performed using the density functional theory (DFT) as implemented in the Gaussian 16 software suite [64]. The B97D3 functional was used, which implemented the Grimme dispersion correction (D3). Transition metal electrons were treated using the SDD basis set; all other electrons were treated using the 6-31G** basis set. Solvation effects were applied using the SMD model with acetic acid (and tetrahydrofuran, where specified) as the implicit medium. No explicit solvent molecules were included in the calculations except for one acetate molecule to simulate its role in the mechanism. Calculations were performed at standard state (1.00 atm, 298.15 K). Frequency calculations (for ground states and transition states) and intrinsic reaction coordinate (IRC) calculations (for transition states only) were performed to verify optimizations. Optimized geometries and thermochemical values are provided in the Supplementary Materials (as a single .xyz file and Tables S2 and S3). Three-dimensional renderings of structures were prepared using CYLview [65] for figures and ChemCraft [66] for animations.

## Figures and Tables

**Table 1:** Atomic distances, bond angles and activation energies for **TS2** (*S*) and (*R*) structures, relative to their starting states in acetic acid and tetrahydrofuran.

Solvent	Structure	d(Ir-H^−^) (Å)	d(C-H^−^) (Å)	d(Ir-H_xyl_) (Å)	∠(C-H_xyl_-Ir) (°)	ΔG^‡^ (kcal mol^−1^)
HOAc	**TS2** (*S*)	1.67	1.76	2.56	150.5	22.7
HOAc	**TS2** (*R*)	1.67	1.79	2.47	136.6	24.0
THF	**TS2** (*S*)	1.69	1.64	2.55	158.9	22.8
THF	**TS2** (*R*)	1.70	1.68	2.47	138.3	25.5
HOAc	**TS2** (*S’*)	1.68	1.64	2.51	124.1	22.7

## Data Availability

The optimized geometries and thermochemical values analyzed in this study are publicly available in the Supplementary Materials.

## Supplementary Materials

The following Supplementary Materials can be downloaded at: https://www.mdpi.com/article/10.3390/molecules27165106/s1, Figure S1: Structure of anionic iridium complex **B**–**I^−^**; Figure S2: Instances of Ir-I (octahedral) atomic distances; Scheme S1: Isomerization of **Im**; Table S1: *E/Z* isomerization equilibrium values; Figure S3: Ground-state optimized structures of imine-acid adducts; Figure S4: Alternative intermediates; Figure S5: Alternative dihydrogen splitting transition states; Figure S6: Alternative hydride transfer transition states; Tables S2–S4: Thermochemical values; animation of the imaginary frequency of **TS1**; animation of the imaginary frequency of **TS2** (*S*); coordinates for all optimized geometries in a single. xyz file. Refs. [64,67,68] are cited in the Supplementary Materials.
