# Supplementary material for: A Plausible Mechanism for the Iridium-Catalyzed Hydrogenation of a Bulky N-Aryl Imine in the (S)-Metolachlor Process
Source: Molecules. 2022 Aug 11;27(16):5106. doi: 10.3390/molecules27165106 (PMC9414898; doi:10.3390/molecules27165106)
Supplement: Supplementary file 1 [file molecules-27-05106-s001.zip › metolachlor_mdpi_SI_july17.pdf]

Supplementary Materials

A Plausible Mechanism for the Iridium-Catalyzed Hydrogenation of a Bulky *N*-Aryl Imine in the (*S*)-Metolachlor Process

Amanda L. Kwan [1] and Robert H. Morris [1,*]

1 Department of Chemistry, University of Toronto, Toronto, ON M5S 3H6, Canada

* Correspondence: rmorris@chem.utoronto.ca

Table of Contents

General Comments 2

Isomerization Calculation 3

Proton Transfer Equilibrium 4

Other Structures and Pathways 4

Alternative Intermediates 4

Alternative Transition States – TS1 (Dihydrogen Splitting) 5

Alternative Transition States – TS2 (Hydride Transfer) 5

Thermochemical Values 7

Optimized Geometries 8

References 9

General Comments

All calculations reported in the main text were performed as described in the Materials and Methods section using Gaussian 16 [1]. Some results reported in the Supplementary Materials include preliminary calculations that were performed using different methods, including the use of Gaussian 09 [2]. For this reason, the version of Gaussian, functional, basis set(s), and implicit solvents will be denoted alongside all thermochemical values reported herein.

All calculations in the main text and Supplementary Materials employed an ultrafine integration grid (accuracy 10^{-11}) and the SMD model of acetic acid or tetrahydrofuran. Calculations were performed at standard state (298.15 K, 1.00 atm). Frequency calculations were performed to validate all stationary states and transition states. Intrinsic reaction coordinate (IRC) calculations were performed to validate main text transition states, including **TS1**, and **TS2** (*S*), and **TS2** (*R*). No corrections to the Gaussian output values were applied. Various corrections have been proposed in the literature (e.g. 1.89 kcal/mol correction to move to 1 M solutions) [3] but these move the Gibbs energy values systematically so that the lowest energy pathway remains the lowest despite shifts in some of the values. Leaving the output uncorrected allows a clear picture of what has been done and will allow others to correct the values when they are better justified.

Iridium-Iodide Distances

An anionic iridium complex **B-I**$^-$ (Figure S1) was calculated using Gaussian 16, functional B97D3, and basis sets SDD (for Fe, Ir, I) and 6-31G** (for all other atoms) in acetic acid as the solvent. The Ir-I distance of the computed complex was compared to Ir-I distances of complexes characterized by X-ray crystallography. Instances reported to the Cambridge Structural Database are represented in Figure S2.

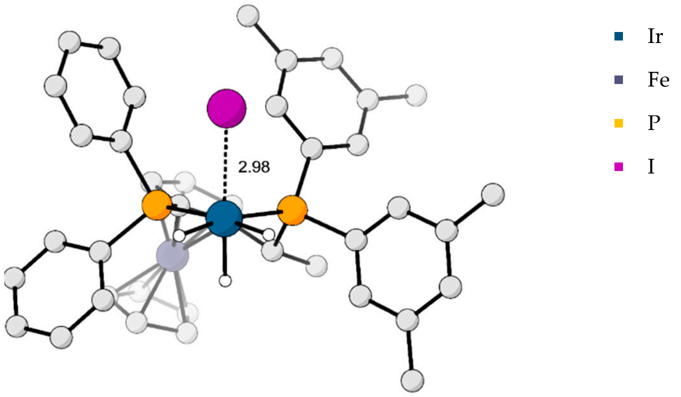

Figure S1. Structure of anionic iridium complex **B-I**$^-$, $[IrH_3LI]^-$ with Ir-I distance reported in Å. C-H hydrogens were omitted for clarity.

According to the current data available, Ir-I bond lengths range from 2.60 to 2.90 Å. The Ir-I distance for **B-I**$^-$ was 2.98 Å, well outside of the known range of Ir-I bond lengths. With this, we justify that iodide can be treated as a weakly- to non-coordinating anion in solution that can be omitted from calculations.

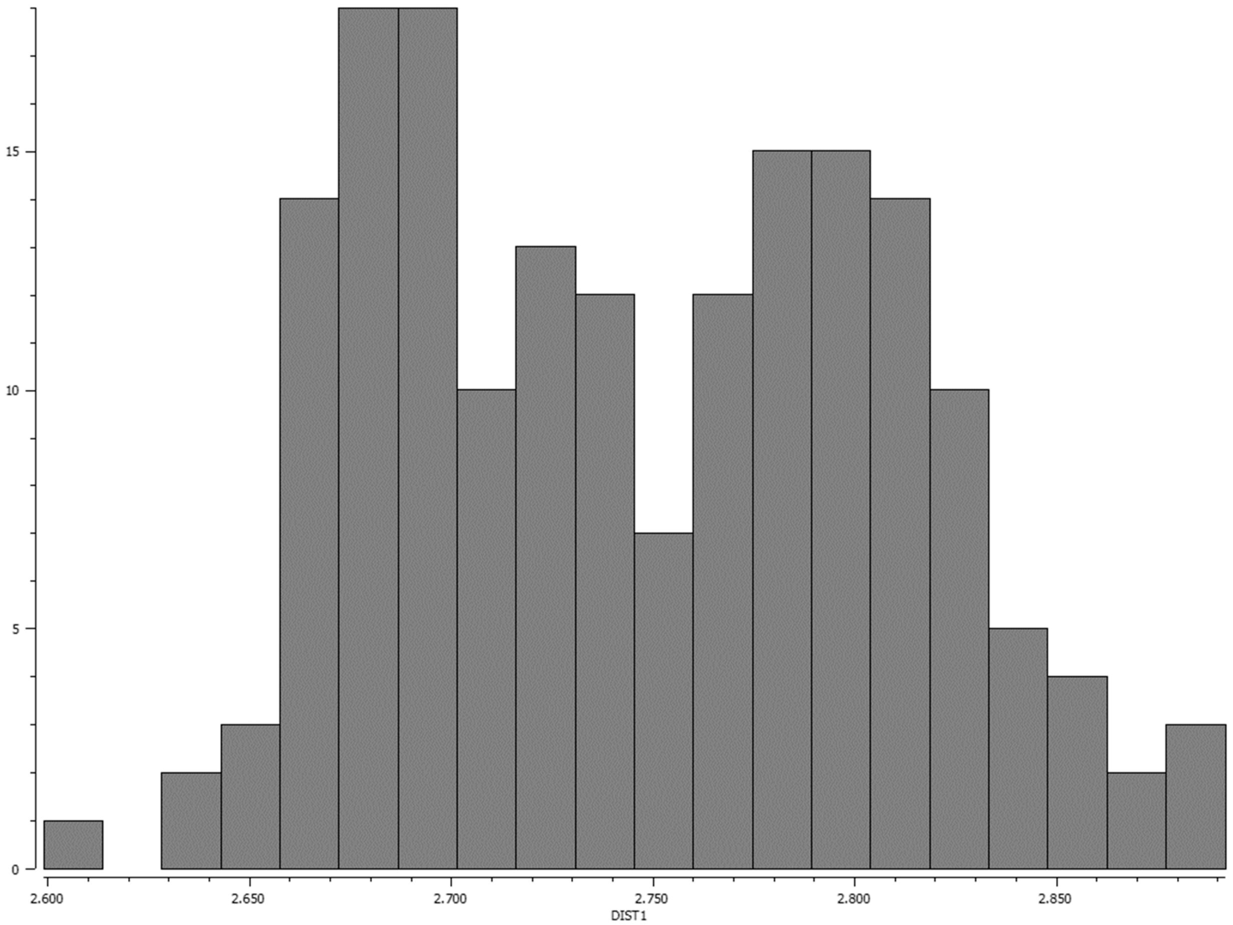

Figure S2. Histogram: Instances of Ir-I (octahedral) atomic distances reported to the Cambridge Structural Database (updated 2022).

Isomerization Calculation

The equilibrium constant K_{eq} was calculated using the free energies of (*E*)-**Im** and (*Z*)-**Im** as defined by Scheme S1. These values were used to calculate the E/Z ratios shown in Table S1.

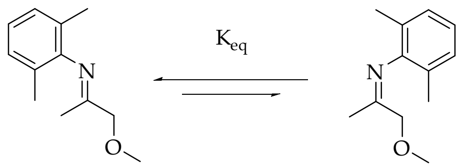

(*E*)-**Im** (*Z*)-**Im**

Scheme S1. Isomerization of **Im**, *N*-(2,6-dimethylphenyl)-1-methoxypropan-2-imine.

Table S1. *E*/*Z* isomer equilibrium values[a] calculated using Gaussian 16, functional B97D3, and basis sets[b] SDD/6-31G** in acetic acid as the solvent.

Solvent	ΔG_{E-Z} (kcal mol^{-1})	K_{eq}	*E*/*Z*
Acetic Acid	1.0648675	0.165747393	86:14
Tetrahydrofuran	1.524825	0.076259775	93:7

[a] T = 273.15 K. [b] SDD for Fe, Ir, and I, 6-31G** for all other atoms.

Proton Transfer Equilibrium

Ground state distances of the isolated adduct Im-H-OAc (Figure S3) show that the proton has a greater affinity towards the stronger base (-OAc); the O-H bond is elongated compared to HOAc (0.98 Å) and the N-H bond is shorter than that of free iminium (1.02-1.03 Å).

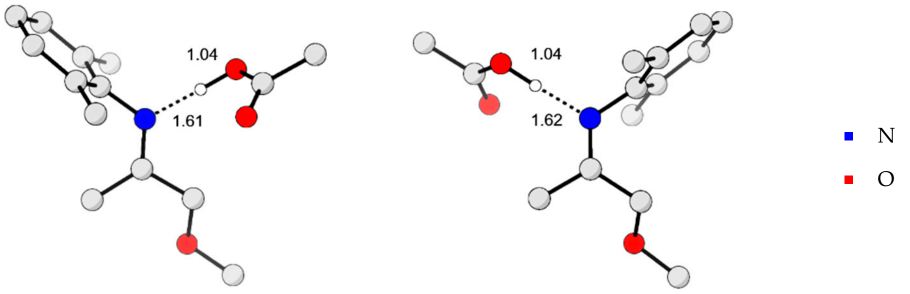

(*E*)-Im-H-OAc (*Z*)-Im-H-OAc

Figure S3. Ground-state optimized structures of imine-acid adducts in acetic acid with distances reported in Å. C-H hydrogens were omitted for clarity.

Other Structures and Pathways

Alternative Intermediates

Other structures that were excluded from or unlabeled in the main text were depicted in Figure S4. Complexes **γ**, **δ**, and **ε** were compared in Figure 7 of the main text. Complex **ζ** was an acetate-free alternative intermediate shown in Figure 10 of the main text. Complexes **θ**, **λ**, **ν**, **ξ**, and **o** were used as reference (starting) states for preliminary calculations reported herein.

Thermochemical values for complexes **γ**, **δ**, **ε**, and **ζ** were reported in Table S2. Thermochemical values for complexes **θ**, **λ**, **ν**, **ξ**, and **o** were reported in Table S4.

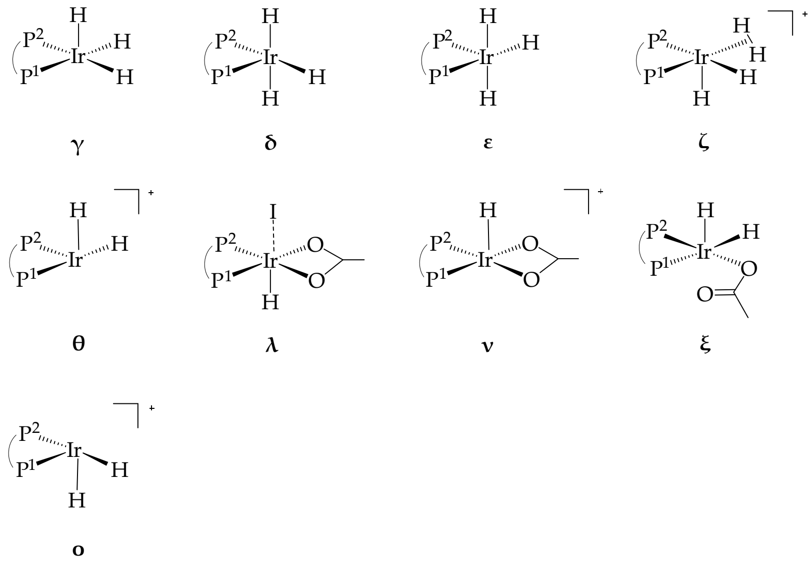

Figure S4. Alternative intermediates.

Alternative Transition States – TS1 (Dihydrogen Splitting)

TS^{III} (an outer-sphere pathway) and **TS^{IV}** (a side-on "catcher's mitt" pathway) shown in Figure S5 were excluded in preliminary calculations due to their large ΔG‡ values.

Thermochemical values and negative frequencies for **TS^{III}** and **TS^{IV}** were reported in Table S4.

Note: Since a different method was used, these values cannot be compared externally to the ΔG‡ of **TS1** reported in the main text.

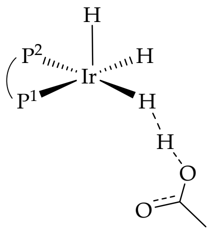

TS^{III} (+ **Im-H$^+$**)
+33.3 kcal mol^{-1} relative to H_2 + HOAc + **Im** + **θ**
Gaussian 09, M11L, SDD/6-31G*, THF

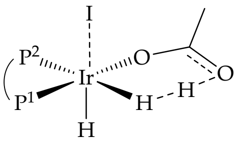

TS^{IV} (+ **Im**)
+19.2 kcal mol^{-1} relative to H_2 + **Im** + **λ**
Gaussian 09, M11L, SDD/6-31G*, THF

Figure S5. Alternative dihydrogen splitting transition states.

Alternative Transition States – TS2 (Hydride Transfer)

Hydride transfer mechanisms from other complexes were explored and shown in Figure S6. **TS^{V}** from a neutral iridium dihydride **ν** and **TS^{VI}** from the other iridium fac-trihydride γ were excluded in preliminary calculations due to their large ΔG‡ values. Hydride transfer mechanisms without acetate were also explored: **TS^{VII}** from γ and **TS^{VIII}** and **TS^{IX}** from **B** were also excluded on the basis of their large ΔG‡ values.

Thermochemical values and negative frequencies for **TS^{V}**, **TS^{VI}**, **TS^{VII}**, **TS^{VIII}**, and **TS^{IX}** were reported in Table S4.

Note: Since a different method was used, these values cannot be compared externally to the ΔG‡ of **TS2** reported in the main text.

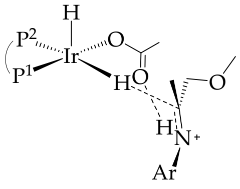

TS^{V} (*S*)
+47.2 kcal mol^{-1} relative to H_2 + **Im** + ν
Gaussian 09, M11L, SDD/6-31G*, THF

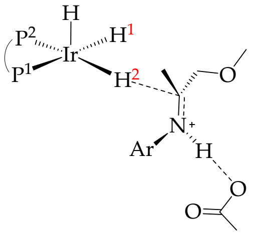

TS^{VI} (*S*) - 98% *ee*
+56.3 kcal mol^{-1} relative to H_2 + **Im** + ξ
Gaussian 09, M11L, SDD/6-31G*, THF

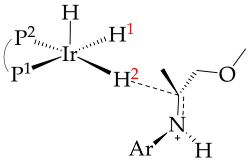

TS^{VII} (*S*) - 53% *ee*
+36.5 kcal mol^{-1} relative to H_2 + **Im** + θ
Gaussian 09, M11L, SDD/6-31G*, THF

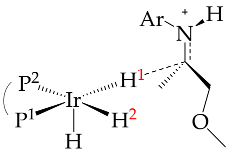

TS^{VIII} (*S*)
+37.6 kcal mol^{-1} relative to H_2 + **Im** + o
Gaussian 09, M11L, SDD/6-31G*, THF

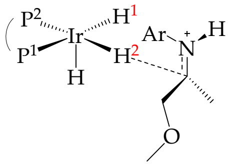

TS^{IX} (*S*) - 57% *ee*
+40.3 kcal mol^{-1} relative to H_2 + **Im** + o
Gaussian 09, M11L, SDD/6-31G*, THF

Figure S6. Alternative hydride transfer transition states.

Thermochemical Values

Table S2. Thermochemical values calculated using Gaussian 16, functional B97D3, and basis sets[a] SDD/6-31G** in acetic acid as the solvent.

Structure	H (Hartrees)	S (cal mol^{-1} K^{-1})	G (Hartrees)	TS Frequency (*i* cm^{-1})
(*E*)-**Im**	-596.844868	127.461	-596.905429	
(*Z*)-**Im**	-596.843988	125.741	-596.903732	
(*E*)-**Im**-H-OAc	-825.762274	161.234	-825.838882	
(*Z*)-**Im**-H-OAc	-825.760823	160.259	-825.836967	
Amine	-598.038092	124.721	-598.097351	
H_2	-1.168381	31.141	-1.183178	
**A**	-2687.894796	289.721	-2688.032452	
**B**	-2460.145317	270.954	-2460.274056	
**TS1**	-2689.063682	292.972	-2689.202882	430.8203
**TS2** (*S*)	-3285.914037	359.700	-3286.084942	252.6929
**TS2** (*R*)	-3285.914100	355.155	-3286.082845	227.9785
**TS2** (*S′*)	-3285.913959	359.843	-3286.084932	208.5713
**B-I$^-$**	-2471.771389	275.059	-2471.902078	
γ	-2460.141582	267.482	-2460.268671	
**δ**	-2460.129848	266.864	-2460.256644	
ε	-2460.129641	266.034	-2460.256043	
**ζ**	-2460.592042	257.103	-2460.714200	

[a] SDD for Fe, Ir, and I, 6-31G** for all other atoms.

Table S3. Thermochemical values calculated using Gaussian 16, functional B97D3, and basis sets[a] SDD/6-31G** in tetrahydrofuran as the solvent.

Structure	H (Hartrees)	S (cal mol^{-1} K^{-1})	G (Hartrees)	TS Frequency (*i* cm^{-1})
(*E*)-**Im**	-596.849475	127.914	-596.910250	
(*Z*)-**Im**	-596.847921	126.070	-596.907820	
Amine	-598.041773	125.239	-598.101278	
H_2	-1.168385	-1.183181	-1.183181	
**A**	-2687.905432	287.544	-2688.042054	
**B**	-2460.158890	272.110	-2460.288178	
**TS2** (*S*)	-3285.926335	363.844	-3286.099209	226.8761
**TS2** (*R*)	-3285.925859	355.608	-3286.094819	303.0063

[a] SDD for Fe, Ir, and I, 6-31G** for all other atoms.

Table S4. Thermochemical values calculated using Gaussian 09, functional M11L, and basis sets[a] SDD/6-31G* in tetrahydrofuran as the solvent.

Structure	H (Hartrees)	S (cal mol^{-1} K^{-1})	G (Hartrees)	TS Frequency (*i* cm^{-1})
(*E*)-**Im**	-597.041254	125.386	-597.100829	
(*E*)-**Im**-H^+	-597.477395	123.826	-597.536229	
H_2	-1.160838	31.135	-1.175631	
HOAc	-228.947696	67.998	-228.980004	
**B**	-2460.355670	264.643	-2460.481411	
**θ**	-2459.628336	259.296	-2459.751536	
**λ**	-2699.110934	291.085	-2699.249238	
**ν**	-2687.438819	279.153	-2687.571453	
**ξ**	-2688.160482		-2688.295659	
**ο**	-2459.634901	262.762	-2459.759748	
**TS^{III}**	-2689.282082	287.634	-2689.418746	239.2233
**TS^{IV}**	-2700.253535	296.096	-2700.394220	1159.7231
**TS^{V}** (*S*)	-3285.610367	341.543	-3285.772645	500.3773
**TS^{VI}** (*S*)	-3286.312965	356.647	-3286.482419	494.8763
**TS^{VI}** (*R*)	-3286.306223	361.326	-3286.477900	647.9208
**TS^{VII}** (*S*)	-3057.811943	332.136	-3057.969752	667.5504
**TS^{VII}** (*R*)	-3057.808988	336.015	-3057.968639	520.8097
**TS^{VIII}** (*S*)	-3057.820240	328.578	-3057.976359	512.4599
**TS^{IX}** (*S*)	-3057.815917	328.525	-3057.972010	623.6236
**TS^{IX}** (*R*)	-3057.813648	330.710	-3057.970779	369.2004

[a] SDD for Fe, Ir, and I, 6-31G* for all other atoms.

Optimized Geometries

Coordinates are available in a single .xyz file. Structures were optimized at the B97D3, SDD/6-31G** level of theory in acetic acid as the solvent unless otherwise specified.

References

[1] Frisch, M.J.; Trucks, G.W.; Schlegel, H.B.; Scuseria, G.E.; Robb, M.A.; Cheeseman, J.R.; Scalmani, G.; Barone, V.; Petersson, G.A.; Nakatsuji, H.; et al. *Gaussian 16*, Revision C.01; Wallingford, CT, USA, 2016.

[2] Frisch, G.W.T.; Schlegel, H. B.; Scuseria, G. E.; Robb, M. A.; Cheeseman, J. R.; Scalmani, G.; Barone, V.; Petersson, G. A.; Nakatsuji, H.; Li, X.; et al. *Gaussian 09*, Revision E.01; Wallingford, CT, USA, 2016.

[3] Harvey, J.N.; Himo, F.; Maseras, F.; Perrin, L. Scope and Challenge of Computational Methods for Studying Mechanism and Reactivity in Homogeneous Catalysis. *ACS Catal.* **2019**, *9*, 6803–6813. https://doi.org/10.1021/acscatal.9b01537.
