# Supplementary figures and images for: A Plausible Mechanism for the Iridium-Catalyzed Hydrogenation of a Bulky N-Aryl Imine in the (S)-Metolachlor Process
Source: Molecules. 2022 Aug 11;27(16):5106. doi: 10.3390/molecules27165106 (PMC9414898; doi:10.3390/molecules27165106)

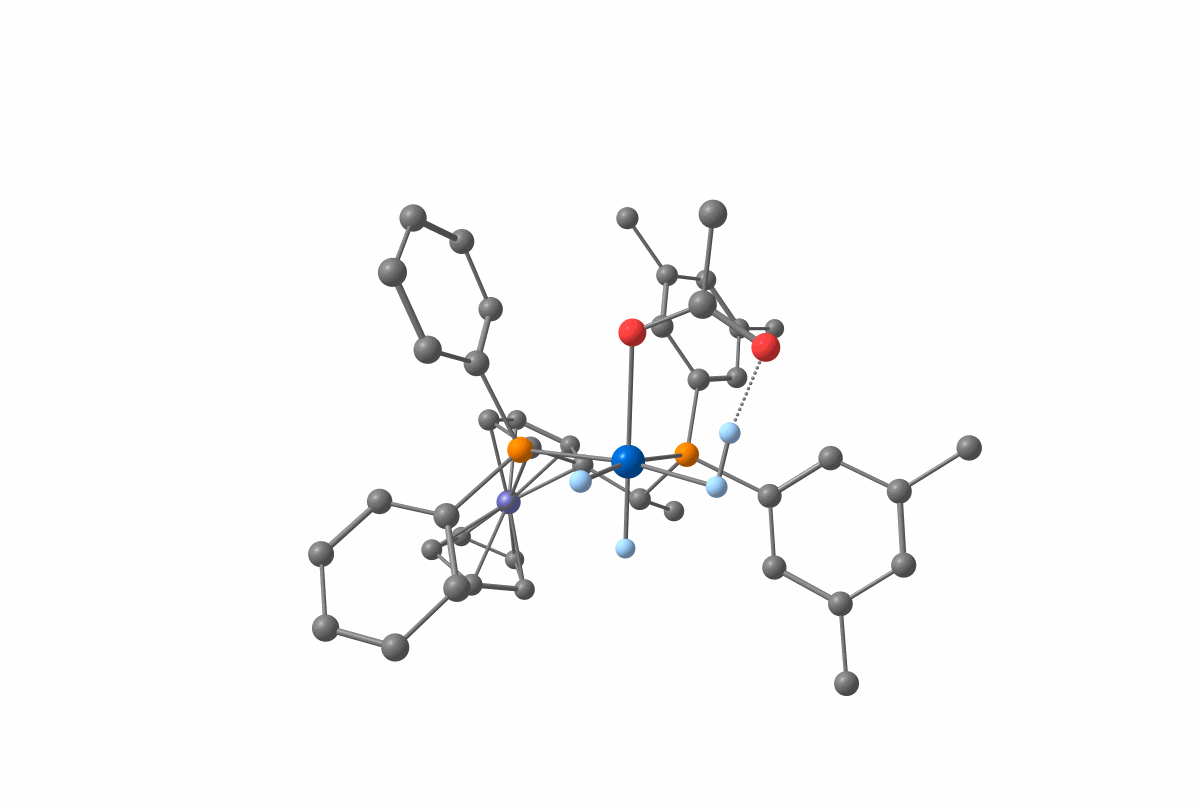

Supplement: Supplementary file 1 [file molecules-27-05106-s001.zip › TS1animated.gif]

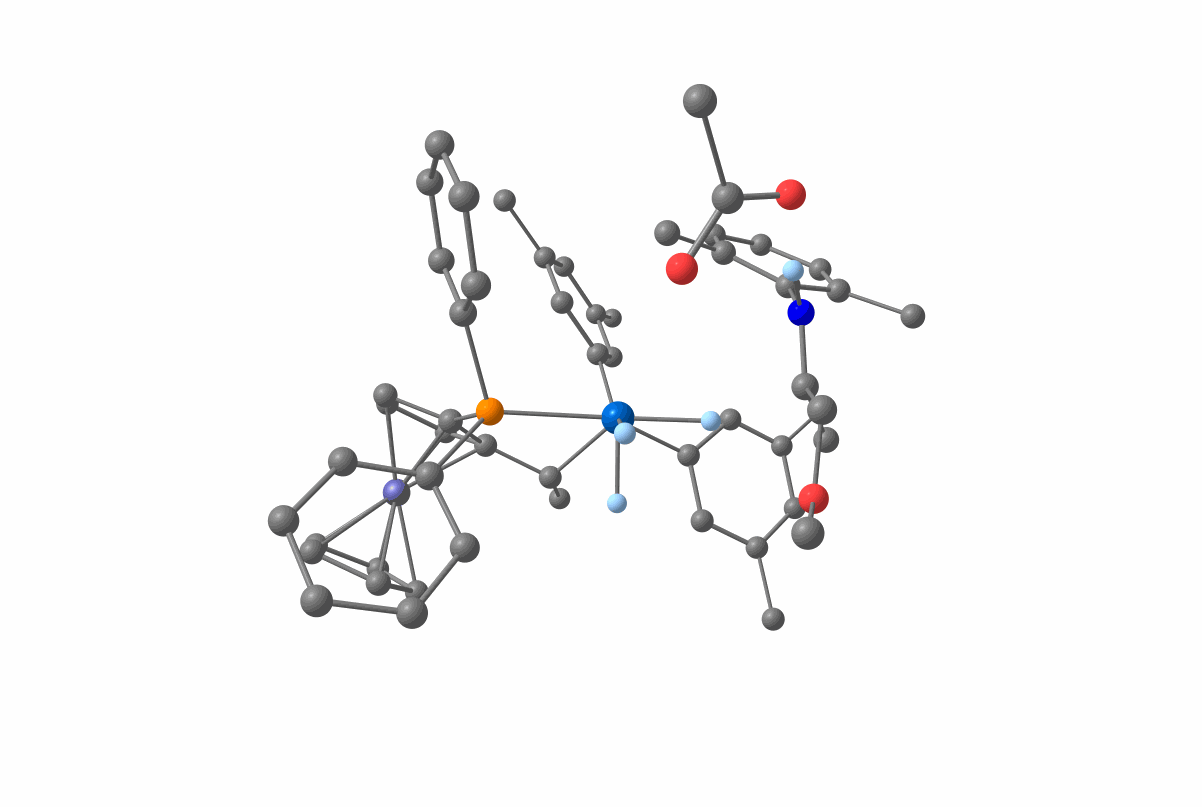

Supplement: Supplementary file 1 [file molecules-27-05106-s001.zip › TS2animated.gif]
